# Interleukin-6 and Leukocyte Cell Population Data in Newly Diagnosed Sepsis—A Prospective Study

**DOI:** 10.3390/medicina61030468

**Published:** 2025-03-07

**Authors:** Sara Šundalić, Iva Košuta, Ivana Baršić Lapić, Ivana Rako, Dunja Rogić, Radovan Radonić, Ana Vujaklija Brajković

**Affiliations:** 1 Department of Internal Medicine, Division of Intensive Care Medicine, University Hospital Centre Zagreb, Kišpatićeva 12, 10000 Zagreb, Croatia; ivakosuta@gmail.com (I.K.); rradonic@gmail.com (R.R.); avujaklija@gmail.com (A.V.B.); 2 Department of Laboratory Diagnostics, University Hospital Centre Zagreb, Kišpatićeva 12, 10000 Zagreb, Croatia; ivana.barsic@kbc-zagreb.hr (I.B.L.); ivana.rako@kbc-zagreb.hr (I.R.); dunja.rogic@kbc-zagreb.hr (D.R.); 3 Department of Medical Biochemistry and Hematology, Faculty of Pharmacy and Biochemistry, University of Zagreb, A. Kovačića 1, 10000 Zagreb, Croatia; 4 School of Medicine, University of Zagreb, Šalata 3, 10000 Zagreb, Croatia

**Keywords:** sepsis, interleukin-6, leukocyte cell population data, neutrophil reactivity intensity, neutrophil granularity intensity, sepsis-associated acute kidney injury, immunocompromised patient, biomarkers

## Abstract

*Background and Objectives:* Sepsis still represents a syndrome with a high mortality. A timely sepsis diagnosis and an early intervention are crucial for the disease outcomes. Sepsis-associated acute kidney injury (SA-AKI) is highly prevalent but often diagnosed late. We aimed to investigate whether serum interleukin-6 (IL-6) and leukocyte cell population data (CPD) could be adequate biomarkers for the prediction of survival and SA-AKI development. *Materials and Methods:* We conducted a prospective observational study in a medical intensive care unit of a tertiary hospital centre in Zagreb, Croatia from June 2020 to October 2023. Adult patients with newly diagnosed sepsis were included and classified as immunocompetent or immunocompromised. Blood samples were collected upon admission. *Results:* A total of 150 patients were included in the study. Ninety-six (64%) patients were immunocompetent and fifty-four (36%) were immunocompromised. The median SOFA score was 8 (6–11). SA-AKI was diagnosed in 108 (72%) patients. ICU and hospital mortality was 27.3% and 37.3%, with no significant difference between groups. Significantly higher serum IL-6 levels were noted in the immunocompromised group, while neutrophil granularity intensity was higher in the immunocompetent group. According to logistic regression analyses, elevated IL-6 levels predicted a lethal ICU outcome, while elevated IL-6 levels and neutrophil reactivity intensity were predictors of SA-AKI development. A cluster analysis revealed two patient groups with different IL-6 concentrations, and further studies indicated that the group with higher IL-6 values had significantly higher SA-AKI occurrence and increased lethal outcomes. *Conclusions:* An early serum IL-6 measurement regardless of the patients’ immune status indicates disease severity. Its measurement in the early phase of disease presentation, potentially in the emergency department, might facilitate ICU admission. Further research is warranted in the field of leukocyte CDP application.

## 1. Introduction

Sepsis is still a syndrome that carries a large mortality rate worldwide [1,2,3,4], contributing to 19.7% of all global deaths [1]. Sepsis mortality is approximately 27% for all hospitalized patients and increases to up to 42% when sepsis is treated in the intensive care unit (ICU) [2]. The “Third International Consensus Definitions for Sepsis and Septic Shock” from 2016 defined sepsis as a life-threatening state due to a dysregulated host response to infection [5]. Time of sepsis diagnosis, type of infection, and patient comorbidities all play a role in survival. Sepsis-induced organ failure is common, but can be overlooked. In the critically ill, sepsis is the most common cause of acute kidney injury (AKI) [6,7,8,9]. The 28th Acute Disease Quality Initiative (ADQI) workgroup defined sepsis-associated acute kidney injury (SA-AKI) as the presence of both sepsis [5] and AKI criteria [6] occurring within seven days of sepsis diagnosis [7]. SA-AKI is probably a result of multiple deleterious events that occur during sepsis [10]. The reported prevalence of SA-AKI varies from <1% to 66% [8].

Interleukin-6 (IL-6) has been studied as an early marker of sepsis and AKI. High values of IL-6 might serve as a good predictor of sepsis mortality [11,12]. Also, in patients with AKI regardless of etiology, higher IL-6 levels at ICU admission predicted worse short-term renal function and an increased mortality [13]. The results of the PROWESS study showed that increased levels of IL-6 were a risk factor for AKI in patients with sepsis, emphasizing the role of inflammation in its development [14]. IL-6 measurement is more accessible after the recent COVID-19 pandemic [15,16,17,18]. Also, the emergence of chimeric antigen receptor (CAR) T-cell therapy and other forms of modern immunotherapy that can result in cytokine release syndrome made the idea of quick point-of-care IL-6 measurements a little more feasible [19].

Morphological changes in activated leukocytes depicted through leukocyte cell population data (CPD) could be viewed as an even earlier biomarker than the biochemical early biomarkers of sepsis such as IL-6. When our body encounters a certain pathogen, the innate cellular immunity is promptly activated. Neutrophils and monocytes change their morphology and function in an attempt to localize the infection. Nowadays, hematology analyzers can provide information about a specific white blood cell’s size, fluorescence (cell’s metabolic activity), and internal structure summarized in numerical data. This additional information is readily available, without the need for more blood sampling, or for the manual revision of blood smears [20]. In the past decade, we witnessed an increase in researchers’ interest in the clinical implementation of leukocyte CPD [21,22,23,24,25,26,27,28,29,30,31,32,33,34,35]. The results are diverse, with some authors acknowledging the usefulness of these data for detecting early sepsis or for distinguishing infection from malignant disease [20,21,26], while others found that it did not aid in assessing disease severity or predicting outcomes [24,25,34]. Currently, a lack of standardization is a significant downfall to this interesting diagnostic niche [20]. Given the two technologies currently in use (Coulter and Sysmex), CPD parameters cannot be used interchangeably. Additionally, the lack of harmonization among different instruments and laboratories, as well as less analytical quality specifications, are notable limitations [20]. However, given the potential of these parameters, efforts have been made toward CPD harmonization between Sysmex XN modules, as demonstrated by Seghezzi et al. [36]. Nonetheless, further improvement and research in this field is warranted.

Nowadays, the number of immunocompromised patients is increasing. These patients are at a higher risk of a complicated course of infection [37,38]. We are facing a challenge distinguishing the patients who would benefit from an earlier ICU admission from those who would never need it. Azoulay et al. demonstrated a significant survival benefit from early ICU admission for patients with hematologic malignancies [39]. A comprehensive physical examination combined with a thorough patient history remains a cornerstone in the decision-making process, but laboratory findings such as lactate levels, biomarkers of inflammation, and specific organ dysfunction add to the overall clinician’s impression of the patient’s disease severity. Considering the limited capacities of ICUs, incorporating additional data that would help clinicians identify which patients could benefit more from ICU settings in the early course of sepsis could somewhat ease the decision. The value of IL-6 in immunocompromised patients with sepsis has been studied to some degree [40], with recent research mainly focused on the pediatric population [41,42,43]. In the adult population with sepsis, immunocompromised patients accounted for a small proportion of the included population [12,44,45]. Moreover, leukocyte CPD studies involving immunocompromised patients with sepsis are scant [46,47].

Our work aimed to investigate whether serum IL-6 or leukocyte CPD could be adequate biomarkers for survival prediction and SA-AKI development in immunocompetent and immunocompromised patients with newly diagnosed sepsis admitted to the ICU and, therefore, aid the clinicians’ assessment of sepsis severity.

## 2. Materials and Methods

### 2.1. Study Design

We conducted a prospective observational study in a medical ICU of the University Hospital Centre Zagreb, Croatia, from June 2020 to October 2023.

Adult patients hospitalized in the ICU because of newly diagnosed sepsis were considered for inclusion. Only the patients who gave their informed consent were included (if the patients were not able to give their consent, it was attained from their legal representatives). Exclusion criteria were pregnancy, administration of more than one dose of an empirical antibiotic before ICU admission, and chronic therapy with IL-6 inhibitors. COVID-19-positive patients were also excluded from the study.

Sepsis was defined according to the “Third International Consensus Definitions for Sepsis and Septic Shock” as a suspected infection and an acute rise in patients’ SOFA score (Sequential Organ Failure Assessment), by 2 points or more [5]. The AKI diagnosis was made according to KDIGO guidelines, including both serum creatinine and/or urine output criteria [6]. SA-AKI was defined according to the ADQI 28 Workgroup definition as the presence of both sepsis and AKI criteria occurring within seven days of sepsis diagnosis [7].

Patients were divided into two groups according to their immunological status (immunocompetent and immunocompromised) before the indexed hospitalization. A patient was considered immunocompromised if they had one or more of the following: neutropenia (neutrophil count < 1 × 10^9^/L), human immunodeficiency virus (HIV) infection, glucocorticoid therapy (>0.5 mg/kg > 30 days) and/or immunosuppressive and/or cytotoxic therapy, recipient of solid organ or hematopoietic stem cell transplant (allogenic or autologous), active malignant disease (hematological or other) [48]. Patients were considered cured of malignant disease after five or more years of remission.

Patients’ demographic data and comorbidities were gathered at admission to the medical ICU. Disease severity scores (SOFA, Acute Physiology and Chronic Health Evaluation II (APACHE II), and Simplified Acute Physiology Score II (SAPS II)) were calculated at admission. Blood samples were collected within the first 24 h after admission to the ICU. A complete blood count including leukocyte CPD (immature granulocytes (IG), total reactive lymphocytes (RE-LYMP), antibody-synthesizing lymphocytes (AS-LYMP), neutrophil granularity intensity (NEUT-GI), neutrophil reactivity intensity (NEUT-RI)), routine biochemistry parameters (glucose, bilirubin, urea, creatinine, C-reactive protein (CRP), procalcitonin (PCT)), lactate and IL-6 levels were determined for all included patients. The complete blood count and leukocyte CPD analysis were performed using the Sysmex XN-3000 hematology analyzer. Blood samples for IL-6 level determination were promptly transported to the laboratory, centrifuged at 3500 rpm for 15 min, separated into two aliquots, and stored at a temperature of −20 °C until analysis. Biochemical parameters (glucose, bilirubin, urea, creatinine, CRP, PCT) and IL-6 values were assessed using Roche Hitachi Cobas cee6000 (Roche, Mannheim, Germany). IL-6 levels were obtained using an electrochemiluminescence method. Blood lactate levels were obtained using a point-of-care blood gas analyzer Gem Premier 3500 (Instrumentation Laboratory, Bedford, MA, USA). All laboratory data, except for IL-6 levels, were immediately available for the attending physicians.

### 2.2. Statistical Analysis

A statistical analysis was performed using the IBM SPSS Statistics 29.0.1.0. software. A normality analysis was carried out using the Shapiro–Wilk test, while the homogeneity of variances between groups was tested using Levene’s test. Appropriate parametric or nonparametric tests were performed. Numerical variables with normal distribution are presented as mean and standard deviation (SD), and those that did not demonstrate normal distribution as median and interquartile range (IQR). Categorical variables are presented through frequency distributions, while their comparison between two patient groups was carried out using the chi-square test. Numerical variables were compared between the two groups using the Student’s *t*-test or Mann–Whiney U-test. A binary logistic regression was used to determine predictors of patients’ outcomes: ICU mortality and SA-AKI development. The usual prerequisites for binary logistic regression analysis were met. A *p* value < 0.05 was considered statistically significant. A power analysis for a binary logistic regression analysis indicated that the minimum sample size to yield a statistical power of 0.90 with an alpha level of 0.05 and an odds ratio of 1.91 (H0 = 0.3, H1 = 0.45) is 146. This analysis was carried out using G*Power for Windows, version 3.1.9.7. This study was approved by the Ethics Committee of the University Hospital Centre Zagreb, Croatia (Class: 8.1-20/25-2 Number: 02/21 AG, date of approval: 24 February 2020) and conducted according to the guidelines of the Declaration of Helsinki.

## 3. Results

### 3.1. Patients’ Characteristics and Outcomes

A total of 150 consecutive patients with sepsis were included, 79 (52.7%) male and 71 (47.3%) female, with a median age of 68.5 years (IQR 56–77). Arterial hypertension, diabetes mellitus, and coronary artery disease were the most frequent comorbidities. The median SOFA score was 8 (6–11). Ninety-six (64%) patients were immunocompetent, while the remaining fifty-four (36%) were immunocompromised (Table 1).

A difference was found in the levels of serum IL-6, which was significantly higher in the immunocompromised group (*p* = 0.02). The NEUT-GI was higher in immunocompetent patients (*p* = 0.03). The immunocompromised patients had more coronary artery diseases (*p* = 0.04), while other demographic data and comorbidities were similar (Table 1).

The immunocompromised group was heterogeneous. The causes of their immunocompromised state are presented in Table 2.

SA-AKI was diagnosed in 108 (72%) patients, while acute renal replacement therapy was needed in 27 (18%) patients. The development of SA-AKI, the need for acute renal replacement therapy, the ICU or hospital length of stay, and the hospital or ICU mortality did not differ between groups (Table 3).

As to microbiology findings, hemoculture-positive sepsis accounted for 77 (51.3%) cases (Gram-negative sepsis in 39 (26%) patients, Gram-positive sepsis in 31 (20.7%) patients, polymicrobial sepsis in 5 (3.3%) and fungal sepsis in 2 (1.3%)). Hemoculture-negative sepsis accounted for the remaining 73 (48.7%) patients. The most common pathogens were *Escherichia coli* and *Klebsiella pneumoniae* followed by *Staphylococcus aureus*. There was no significant difference between the immunocompetent and immunocompromised patient groups regarding the type of sepsis (hemoculture-positive sepsis or hemoculture-negative sepsis) (*p* = 0.179). Also, ICU and hospital mortality did not differ between patients who had hemoculture-positive and those who had hemoculture-negative sepsis (*p* = 0.969; *p* = 1).

### 3.2. Logistic Regression Models and Cluster Analysis

In a logistic regression model in which lethal outcome in the ICU was the dependent variable, while the predictor variables were NEUT-GI, NEUT-RI, IG, IL-6, and the immunological status of the patient was a categorical variable, we found that IL-6 values are a statistically significant predictor of outcome (b = 0.000, SE = 0.000, *p* = 0.000, OR = 1.000), which would mean that a unit change in IL-6 value does enhance the odds of a lethal outcome to a slight extent (Table 4).

In a logistic regression model, in which SA-AKI development in the ICU was the dependent variable, while the predictor variables were NEUT-GI, NEUT-RI, IG, IL-6, and the immunological status of the patient was a categorical variable, we found that IL-6 values are a statistically significant predictor of outcome (b = 0.000, SE = 0.000, *p* = 0.004, OR = 1.000), which would mean that a unit change in IL-6 value enhances the odds of SA-AKI development in the ICU discretely. We also found that NEUT-RI is a statistically significant predictor of SA-AKI development (b = 0.059, SE = 0.022, *p* = 0.007, OR = 1.061), which would mean that a unit change in NEUT-RI would slightly enhance the odds of SA-AKI development (Table 5).

Following the results of the logistic regression analyses, we carried out a cluster analysis which resulted in two groups of patients divided according to their IL-6 serum levels (Table 6). A chi-square test was then performed for the IL-6 clusters and SA-AKI development (chi-square = 12.07, df = 1, *p* < 0.001), and for the IL-6 clusters and ICU mortality (chi-square = 12.59, df = 1, *p* < 0.001). These results implicate that high IL-6 values are strongly associated with SA-AKI development and a lethal ICU outcome.

## 4. Discussion

Our study prospectively included 150 patients with newly diagnosed sepsis. The immunocompetent and immunocompromised groups were well matched according to gender, age, disease severity, and standard inflammatory markers such as CRP and PCT. We observed three major differences between the two groups of patients: the level of IL-6 and NEUT-GI at ICU admission and the presence of coronary artery disease in patients’ history. The patients were severely ill at admission according to the severity scores. The overall ICU mortality was 27.3%, being somewhat higher in the immunocompromised group of patients, but without a significant difference in comparison to the immunocompetent group of patients.

The logistic regression analyses showed that elevated IL-6 levels are a predictor of ICU mortality and SA-AKI development, with high values vastly implying the development of both outcomes according to the additional cluster analyses. IL-6 has, up till now, been researched in both sepsis and acute kidney injury, in various in vitro, animal, and human studies, in children and adults. Numerous studies showed that IL-6 measurements have a place in survival prediction for patients with serious infections [11,12]. Greenhill et al. provided a possible explanation of the relationship between the increased production of IL-6 and sepsis mortality. They demonstrated, in a genetically modified mouse model of Gram-negative septic shock (lipopolysaccharide/Toll-like receptor 4 (LPS/TLR4) mediated septic shock), that IL-6 trans-signalling (involving the soluble sIL-6Rα) exacerbates TLR4-dependent inflammatory responses. These genetically modified mice demonstrated hyperresponsiveness to LPS due to a specific upregulation of IL-6 in a gp130/STAT3- and TLR/Mal-dependent manner, indicating that both pathways promote the production of IL-6 in response to LPS [49]. Experimental animal models also suggest that IL-6 could play an integral part in the development of AKI, as shown by Chen et al., as the TLR4 (−/−) mice did not develop AKI after an ischemic insult compared to TLR4 (+/+) wild-type mice. Only TLR4 (+/+) leukocytes after infiltrating the injured kidney produced IL-6 due to their interaction with high-mobility group protein B1 (HMGB1) released from injured renal cells [50]. IL-6 seems to play a role not just in the kidney, but also in a multiorgan perspective, acting directly or through mediators; for example, in lung injury that develops after AKI [51,52]. In pediatric postcardiac surgery patients, IL-6 levels were higher in patients who developed AKI and high levels were associated with a prolonged period of mechanical ventilation [53]. Urinary IL-6 also seems to be able to distinguish acute from chronic kidney disease, albeit this is a conclusion based on a study carried out in dogs [54]. Our results might contribute to the notion that serum IL-6 measurements, maybe already in the emergency department, could help differentiate the patients who could benefit from earlier ICU admittance and hopefully enhance sepsis survival.

Our research indicated a high incidence of SA-AKI, reaching 72%. Such a high observed incidence of SA-AKI could be mostly explained either by dehydration in the early course of sepsis and the initial low urine output, or accentuated vigilance in the ICU for this organ dysfunction. However, a decreased urine output, especially in the early course of sepsis, may just be a response to sepsis and not necessarily a marker of kidney injury [55]. In the studied population the need for acute renal replacement therapy was rather low (only 18%). This could implicate that we are dealing with different mechanisms of injury on differently capacitated patients, with specific phenotypes, subphenotypes, and endotypes of SA-AKI [7]. Timely preventive strategies for AKI progression were also applied. Some injuries resolve with the restoration of adequate tissue perfusion, others with adequate antimicrobial therapy, discontinuation of nephrotoxic therapy, etc. [56]. As our results showed, serum IL-6 is a predictor of SA-AKI development regardless of the patients’ immune status, which could indicate the direction in which more attention should be pointed towards—recognizing kidney dysfunction and diminishing the risk with preventive measures, such as implementing nephrotoxic stewardship, obtaining adequate volume status, tissue perfusion, etc. [56,57].

Interestingly, only one parameter of the leukocyte CPD was significantly different between the two groups. NEUT-GI, a morphological sign of cell activity, was higher in the immunocompetent group. NEUT-RI, neutrophil reactivity intensity, was also higher in the immunocompetent group, though not statistically significant. This could be interpreted as a sign of an adequate host response to infection. On the other hand, extremely elevated IL-6 levels could point to a maladaptive host response which is more common in the immunocompromised group. Studies in patients with various malignant diseases have shown that high serum IL-6 levels are associated with a poor prognosis and survival rate [58]. However, NEUT-RI was a significant predictor for SA-AKI development regardless of the immune status of the patient. This could indicate that a genetic predisposition for kidney injury could determine whether elevated levels of circulating activated neutrophils cause kidney injury, which could be similar to the conclusions from the earlier studies with TLR (+/+) mice and IL-6 [50]. The pathophysiology of these findings is beyond the scope of this research. Further research regarding cell morphology changes and cytokine production is needed to elucidate the connection between leukocyte activation (specific cell type and specific morphological change), elevated levels of IL-6, and organ-specific damage.

The notion that immunocompromised patients had coronary artery disease more frequently might be explained by chronic corticosteroid use (52% of the immunocompromised patients). Studies from Wei et al. and Souverein et al. indicate that cardiovascular disease is more prevalent in corticosteroid users [59,60]. Specific autoimmune diseases like rheumatoid arthritis and systemic lupus erythematosus are associated with an increased cardiovascular risk [61,62,63]. Moreover, kidney transplant recipients also have an increased cardiovascular risk due to chronic kidney disease [64]. As a possible limitation of this finding, it must be emphasized that immunocompromised patients undergo a thorough cardiological examination before cardiotoxic chemotherapy or major surgery. As patients from the immunocompetent group do not undergo this screening process, they could be underdiagnosed regarding coronary artery disease.

The strength of our study is the relatively large number of included patients with sepsis who were admitted to the ICU early in the course of their disease, together with a prospective study design. Additionally, we included a significant proportion of immunocompromised patients in our study population. Our results could help fill the gap in the current understanding of IL-6 and leukocyte CPD application in the early course of sepsis in both immunocompetent and immunocompromised patients.

The limitations of this study include a heterogeneous population of immunocompromised patients, whose immunocompromised status was determined according to their medical history and chronic therapy but was not based on a specific laboratory finding. Further larger cohort studies are warranted, with emphasis on multi-centre studies, to adequately assess and navigate the clinical implementation of IL-6 and leukocyte CPD in septic patients.

## 5. Conclusions

Sepsis continues to carry a high mortality rate and SA-AKI is its well-known, but often overlooked, companion. Our results demonstrated that the early measurement of serum IL-6 and leukocyte CPD (especially neutrophil-related parameters) are an additional indicator of disease severity, regardless of the patients’ immune state. High IL-6 values were strongly associated with an increased ICU mortality and SA-AKI development. Therefore, an initial measurement of IL-6 in the emergency department could facilitate timely ICU admission for patients with sepsis. Further research is warranted in the field of leukocyte CDP application.

## Figures and Tables

**Table 1:** Patients’ characteristics with a comparison regarding their immunological status.

	Total Population (N = 150)	Immunocompetent Group (N = 96)	Immunocompromised Group (N = 54)	*p* Value
Age (median, IQR)	68.5 (56–77)	70 (55.3–78.8)	65 (57.8–74)	0.91
Male, N (%)	79 (52.7)	48 (50.0)	31 (57.4)	0.48
Arterial hypertension, N (%)	84 (56)	53 (55.2)	31 (57.4)	0.93
Diabetes mellitus, N (%)	44 (29.3)	31 (32.3)	13 (24.1)	0.38
CAD, N (%)	32 (21.3)	15 (15.6)	17 (31.5)	0.04 *
SOFA (median, IQR)	8 (6–11)	8 (5–12)	8 (6–11)	0.85
APACHE II (median, IQR)	22 (17–28)	22 (15–27)	23 (18–29)	0.17
SAPS II (median, IQR)	44 (35–60)	43 (32–57)	50 (38–64)	0.05
CRP (mg/L) (median, IQR)	203 (106–294)	208 (109–310)	197 (103–286)	0.38
PCT (μg/L) (median, IQR)	15.5 (2.1–53.2)	16.5 (2.3–60.8)	6.3 (1.9–39.8)	0.15
IL-6 (pg/mL) (median, IQR)	552 (99–4847)	387 (79–1370)	1796 (180–5001)	0.02 *
IG (%) (median, IQR)	1.4 (0.7–3.6)	1.5 (0.8–3.8)	1.0 (0.5–3.4)	0.29
NEUT-GI (SI) (mean, SD)	154 (±4.9)	154 (±4.9)	152 (±4.6)	0.03 *
NEUT-RI (FI) (median, IQR)	55.5 (50.2–63.6)	56.6 (51.9–64.4)	51.7 (48.8–63.6)	0.07

Legend: CAD coronary artery disease, SOFA Sequential Organ Failure Assessment, APACHE II Acute Physiology and Chronic Health Evaluation II, SAPS II Simplified Acute Physiology Score II, CRP C-reactive protein, PCT procalcitonin, IG Immature Granulocytes, NEUT-GI Neutrophil Granularity Intensity, SI Scatter Intensity, NEUT-RI Neutrophil Reactivity Intensity, FI Fluorescence Intensity, IQR interquartile range, * statistically significant *p* < 0.05.

**Table 2:** Immunocompromised patient group (N = 54) and the causes of the immunocompromised state.

Cause	Prevalence, N (%)
Chronic corticosteroid therapy	28 (52)
Malignant disease (solid organ)	24 (44)
Hematologic malignancy	16 (30)
Solid organ transplantation	4 (7)
Hematopoietic stem cell transplantation *	5 (9)
Autoimmune disease **	10 (19)

Legend: * Hematopoietic stem cell transplantation included autologous (2 (4%)) and allogenic (3 (6%)) transplantation. ** Autoimmune diseases included: 4 (7%) patients with rheumatoid arthritis, 2 (4%) with ANCA positive vasculitis, 1 (2%) with HLA-B27 positive arthritis, 1 (2%) with juvenile arthritis, 1 (2%) with myasthenia gravis and 1 (2%) with Crohn’s disease.

**Table 3:** Patients’ clinical outcomes with comparison regarding immunological status.

	Total Population (N = 150)	Immunocompetent Group (N = 96)	Immunocompromised Group (N = 54)	*p* Value
SA-AKI, N (%)	108 (72)	69 (71.9)	39 (72.2)	1.0
Acute RRT, N (%)	27 (18)	20 (20.8)	7 (13)	0.33
ICU mortality, N (%)	41 (27.3)	21 (21.9)	20 (37)	0.07
Hospital mortality, N (%)	56 (37.3)	31 (32.3)	25 (46.3)	0.139
ICU length of stay, days, median (IQR)	6 (3–11)	7 (3–11)	5 (2.75–8.5)	0.058
Hospital length of stay, days, median (IQR)	13 (7–23.5)	13 (8–22)	10.5 (4–26.5)	0.419

Legend: SA-AKI sepsis-associated acute kidney injury, RRT renal replacement therapy.

**Table 4:** Logistic regression and odds ratio (OR) in predicting ICU mortality.

	b	SE	z	*p*	OR	95% CI
(Intercept)	3.792	5.124	0.740	0.459	44.326	0.002–1,088,750.744
NEUT-GI	−0.045	0.034	−1.350	0.177	0.956	0.894–1.020
NEUT-RI	0.020	0.013	1.590	0.112	1.021	0.995–1.048
IG	−0.016	0.033	−0.488	0.626	0.984	0.915–1.044
IL-6	0.000	0.000	3.813	0.000 *	1.000	1.000–1.001
Immunocompromised patient	0.420	0.355	1.185	0.236	1.522	0.752–3.037

Legend: NEUT-GI Neutrophil Granularity Intensity, NEUT-RI Neutrophil Reactivity Intensity, IG Immature Granulocytes (%), IL-6 Interleukin-6, SE Standard Error, OR Odds Ratio, 95% CI 95% Confidence Interval, * statistically significant *p* < 0.05.

**Table 5:** Logistic regression and odds ratio (OR) in predicting SA-AKI development.

	b	SE	z	*p*	OR	95% CI
(Intercept)	4.577	4.510	1.015	0.310	97.252	0.015–753,888.447
NEUT-GI	−0.048	0.030	−1.590	0.112	0.954	0.898–1.010
NEUT-RI	0.059	0.022	2.703	0.007 *	1.061	1.020–1.111
IG	−0.022	0.031	−0.690	0.490	0.979	0.922–1.046
IL-6	0.000	0.000	2.907	0.004 *	1.000	1.000–1.001
Immunocompromised patient	−0.212	0.329	−0.644	0.520	0.809	0.426–1.552

Legend: NEUT-GI Neutrophil Granularity Intensity, NEUT-RI Neutrophil Reactivity Intensity, IG Immature Granulocytes (%), IL-6 Interleukin-6, SE Standard Error, OR Odds Ratio, 95% CI 95% Confidence Interval, * statistically significant *p* < 0.05.

**Table 6:** Descriptive statistics for the two clusters based on the measured serum IL-6 values.

Cluster	N	Median	IQR
1	41	4972.07	4946.79–4994.83
2	92	180.04	70.14–557.3

Legend: N number of patients in a cluster, IQR interquartile range.

## Data Availability

The raw data supporting the conclusions of this article will be made available by the authors upon request and after additional approval from the Ethics committee is granted.

## Abbreviations

The following abbreviations are used in this manuscript:

ADQI 28	The 28th Acute Disease and Quality Initiative
AKI	Acute kidney injury
APACHE II	Acute Physiology and Chronic Health Evaluation II
AS-LYMP	Antibody-synthesizing lymphocytes
CPD	Cell population data
CRP	C-reactive protein
HMGB1	High-mobility group protein B1
ICU	Intensive care unit
IG	Immature granulocytes
IL-6	Interleukin-6
JAK/STAT	Janus kinase/signal transducer and activator of transcription
KDIGO	Kidney Disease: Improving Global Outcomes
LPS	Lipopolysaccharide
NEUT-GI	Neutrophil granularity intensity
NEUT-RI	Neutrophil reactivity intensity
PCT	Procalcitonin
RE-LYMP	Total reactive lymphocytes
SA-AKI	Sepsis-associated acute kidney injury
SAPS II	Simplified Acute Physiology Score II
SOFA	Sequential Organ Failure Assessment
TLR4	Toll-like receptor 4
